# Dynamic clamping human and rabbit atrial calcium current: narrowing *I*_CaL_ window abolishes early afterdepolarizations

**DOI:** 10.1113/JP277827

**Published:** 2019-06-12

**Authors:** Sarah Kettlewell, Priyanka Saxena, John Dempster, Michael A. Colman, Rachel C. Myles, Godfrey L. Smith, Antony J. Workman

**Affiliations:** ^1^ Institute of Cardiovascular & Medical Sciences University of Glasgow Glasgow UK; ^2^ Strathclyde Institute of Pharmacy & Biomedical Sciences University of Strathclyde Glasgow UK; ^3^ School of Biomedical Sciences University of Leeds Leeds UK

**Keywords:** Dynamic‐clamp, Atrial myocyte, Calcium window current, Afterdepolarisations, Atrial fibrillation

## Abstract

**Key points:**

- Early‐afterdepolarizations (EADs) are abnormal action potential oscillations and a known cause of cardiac arrhythmias. Ventricular EADs involve reactivation of a Ca^2+^ current (*I*_CaL_) in its ‘window region’ voltage range. However, electrical mechanisms of atrial EADs, a potential cause of atrial fibrillation, are poorly understood.
- Atrial cells were obtained from consenting patients undergoing heart surgery, as well as from rabbits. *I*_CaL_ was blocked with nifedipine and then a hybrid patch clamp/mathematical‐modelling technique, ‘dynamic clamping’, was used to record action potentials at the same time as injecting an artificial, modifiable, *I*_CaL_ (*I*_CaL,D‐C_).
- Progressively widening the *I*_CaL,D‐C_ window region produced EADs of various types, dependent on window width. EAD production was strongest upon moving the activation (*vs*. inactivation) side of the window.
- EADs were then induced by a different method: increasing *I*_CaL,D‐C_ amplitude and/or K^+^ channel‐blockade (4‐aminopyridine). Narrowing of the *I*_CaL,D‐C_ window by ∼10 mV abolished these EADs.
- Atrial *I*_CaL_ window narrowing is worthy of further testing as a potential anti‐atrial fibrillation drug mechanism.

**Abstract:**

Atrial early‐afterdepolarizations (EADs) may contribute to atrial fibrillation (AF), perhaps involving reactivation of L‐type Ca^2+^ current (*I*_CaL_) in its window region voltage range. The present study aimed (i) to validate the dynamic clamp technique for modifying the *I*_CaL_ contribution to atrial action potential (AP) waveform; (ii) to investigate the effects of widening the window *I*_CaL_ on EAD‐propensity; and (iii) to test whether EADs from increased *I*_CaL_ and AP duration are supressed by narrowing the window *I*_CaL_. *I*_CaL_ and APs were recorded from rabbit and human atrial myocytes by whole‐cell‐patch clamp. During AP recording, *I*_CaL_ was inhibited (3 µm nifedipine) and replaced by a dynamic clamp model current, *I*_CaL,D‐C_ (tuned to native *I*_CaL_ characteristics), computed in real‐time (every 50 µs) based on myocyte membrane potential. *I*_CaL,D‐C_‐injection restored the nifedipine‐suppressed AP plateau. Widening the window *I*_CaL,D‐C_, symmetrically by stepwise simultaneous equal shifts of half‐voltages (*V*_0.5_) of *I*_CaL,D‐C_ activation (negatively) and inactivation (positively), generated EADs (single, multiple or preceding repolarization failure) in a window width‐dependent manner, as well as AP alternans. A stronger EAD‐generating effect resulted from independently shifting activation *V*_0.5_ (asymmetrical widening) than inactivation *V*_0.5_; for example, a 15 mV activation shift produced EADs in nine of 17 (53%) human atrial myocytes *vs*. 0 of 18 from inactivation shift (*P* < 0.05). In 11 rabbit atrial myocytes in which EADs were generated either by increasing the conductance of normal window width *I*_CaL,D‐C_ or subsequent 4‐aminopyridine (2 mm), window *I*_CaL,D‐C_ narrowing (10 mV) abolished EADs of all types (*P* < 0.05). The present study validated the dynamic clamp for *I*_CaL_, which is novel in atrial cardiomyocytes, and showed that EADs of various types are generated by widening (particularly asymmetrically) the window *I*_CaL_, as well as abolished by narrowing it. Window *I*_CaL_ narrowing is a potential therapeutic mechanism worth pursuing in the search for improved anti‐AF drugs.

## Key points

- Early‐afterdepolarizations (EADs) are abnormal action potential oscillations and a known cause of cardiac arrhythmias. Ventricular EADs involve reactivation of a Ca^2+^ current (*I*_CaL_) in its ‘window region’ voltage range. However, electrical mechanisms of atrial EADs, a potential cause of atrial fibrillation, are poorly understood.
- Atrial cells were obtained from consenting patients undergoing heart surgery, as well as from rabbits. *I*_CaL_ was blocked with nifedipine and then a hybrid patch clamp/mathematical‐modelling technique, ‘dynamic clamping’, was used to record action potentials at the same time as injecting an artificial, modifiable, *I*_CaL_ (*I*_CaL,D‐C_).
- Progressively widening the *I*_CaL,D‐C_ window region produced EADs of various types, dependent on window width. EAD production was strongest upon moving the activation (*vs*. inactivation) side of the window.
- EADs were then induced by a different method: increasing *I*_CaL,D‐C_ amplitude and/or K^+^ channel‐blockade (4‐aminopyridine). Narrowing of the *I*_CaL,D‐C_ window by ∼10 mV abolished these EADs.
- Atrial *I*_CaL_ window narrowing is worthy of further testing as a potential anti‐atrial fibrillation drug mechanism.

## Introduction

Afterdepolarizations are premature action potentials (AP) or subthreshold depolarizations that depend on the preceding AP for their formation (Fozzard, 1992). Early afterdepolarizations (EAD) are transient reversals of AP repolarization that occur either during early (phase 2 EADs) or terminal (late phase 3 EADs) repolarization (Weiss *et al*. 2010). EADs can promote arrhythmias by causing either triggered activity, or electrical heterogeneity which could predispose to unidirectional conduction block and reentrant excitation (Wit & Boyden, 2007; Weiss *et al*. 2010; Colman *et al*. 2017), and they are an established cause of polymorphic ventricular tachycardia in long QT syndrome (Yan *et al*. 2001). Evidence is accumulating that EADs could also be involved in the generation and maintenance of the most common cardiac arrhythmia: atrial fibrillation (AF) (Burashnikov & Antzelevitch, 2003; Wit & Boyden, 2007; Guo *et al*. 2010; Watanabe *et al*. 2010; Numata *et al*. 2012). There is a major unmet clinical need to develop more effective and safe anti‐AF drugs with novel mechanisms of action (Workman *et al*. 2011). Because the suppression of atrial EADs may be a potential therapeutic target, investigations aimed at improving our understanding of the electrophysiological mechanisms of their formation and termination are required.

The dynamic clamp, a powerful hybrid patch clamp/computational modelling technique, is emerging as a tool ideally suited to such investigations (Wilders, 2006; Berecki *et al*. 2014; Ortega *et al*. 2018). EADs result from a complex interaction between membrane voltage (*V*_m_), ion currents, such as L‐type Ca^2+^ (*I*_CaL_), Na^+^/Ca^2+^‐exchanger (*I*_Na/Ca_), late Na^+^ (*I*_NaL_) and delayed rectifier (*I*_Ks_, *I*_Kr_), and intracellular Ca^2+^ (Ca^2+^_i_)‐cycling, although it is exceedingly difficult to dissect these components and study them systematically (Weiss *et al*. 2010; Qu *et al*. 2013). Regenerative reactivation of *I*_CaL_ in the ‘window region’ (where steady‐state *I*_CaL_ activation and inactivation curves overlap), when AP duration (APD) is sufficiently prolonged, is considered a prominent EAD mechanism, as shown in the ventricle (January & Riddle, 1989; Fozzard, 1992; Weiss *et al*. 2010; Qu *et al*. 2013). Dynamic clamping, by injecting into cardiomyocytes a mathematically modelled *I*_CaL_ that changes dynamically according to *V*_m_, permits the investigation of effects on APs and EADs of systematically manipulating the voltage‐dependent characteristics of this window current. Furthermore, because the injected current is not carried by Ca^2+^ ions, this provides a unique opportunity to attempt to separate the electrical effects of *I*_CaL_ from the chemical effects of Ca^2+^ (Berecki *et al*. 2014). Dynamic clamping has been used, in rabbit ventricular myocytes, to show that EADs produced by H_2_O_2_ or low [K^+^]_o_ could be suppressed purely by the electrical effect of either shifting *I*_CaL_ activation or inactivation curves to narrow the window region (Madhvani *et al*. 2011), or decreasing a non‐inactivating, pedestal component of *I*_CaL_ (Madhvani *et al*. 2015). Furthermore, a mathematical model of AP that was optimized after dynamic clamping several ion currents in guinea‐pig ventricular myocytes showed that, when APD was sufficiently prolonged, an increase in *I*_CaL_ conductance could produce EADs suggestive of window *I*_CaL_ reactivation (Devenyi *et al*. 2017).

However, in contrast to ventricle, little is known about the contribution of window *I*_CaL_ to EAD formation in the atrium and, to our knowledge, no study has yet dynamic clamped *I*_CaL_ in atrial myocytes from any species. In rabbit atrial myocytes, we previously observed that the take‐off potential of EADs (produced by decreasing transient outward K^+^ current (*I*_TO_) in the presence of β‐adrenergic stimulation) was consistently close to the *V*_m_ of the *I*_CaL_ window centre (Workman *et al*. 2012). This suggested that atrial EADs could also involve reactivation of window *I*_CaL_. However, whether the propensity to EADs, in either rabbit or human atrial myocytes, could be altered by shifting either *I*_CaL_ voltage‐dependent activation or inactivation, presently remains unknown. Such knowledge would advance our mechanistic understanding of atrial EAD formation and could have a crucial bearing on the potential clinical therapeutic utility of window *I*_CaL_ modification in the treatment of AF. Because the atria of patients with established (chronic) AF have a shortened APD and reduced *I*_CaL_, having undergone electrical remodelling (Workman *et al*. 2008; Heijman *et al*. 2014), this could limit the therapeutic potential of window *I*_CaL_ modification in such patients. However, such a limitation should not be expected in patients with non‐AF‐remodelled atria but who are nevertheless susceptible to new‐onset or paroxysmal AF.

The present study aimed to: (i) validate the dynamic clamp for simulating, in atrial myocytes from rabbits and from patients in sinus rhythm, the electrical contribution of *I*_CaL_ to APs; (ii) investigate the effects of widening the *I*_CaL_ window (symmetrically and asymmetrically) on the propensity to induce EADs; and (iii) test whether EADs produced by increasing the *I*_CaL_ conductance and APD, could be supressed by narrowing the *I*_CaL_ window.

## Methods

### Ethical approval

Procedures and experiments involving human atrial cells were approved by West of Scotland Research Ethics Service (REC: 99MC002, 17/WS/0134). Written, informed consent was obtained from all patients. The investigation conformed with the principles outlined in the *Declaration of Helsinki*. Procedures and experiments involving rabbit atrial cells (UK Project Licences: 60/4206, 70/8835) were approved by Glasgow University Ethics Review Committee, and conformed with the guidelines from Directive 2010/63/EU of the European Parliament on the protection of animals used for scientific purposes. The investigators understand the ethical principles under which the *Journal of Physiology* operates, and our work complies with the animal ethics checklist in (Grundy, 2015).

### Atrial cardiomyocytes

Cardiomyocytes were isolated from rabbit and human atrial tissues, as described previously (Workman *et al*. 2000; Workman *et al*. 2001). Rabbits (New Zealand White [Envigo, Huntington, UK], male, aged 16–34 weeks, weighing 2.1–3.6 kg, feeding *ad libitum*; *n* = 36) were humanely killed by i.v. injection of anaesthetic (100 mg kg^–1^ Na^+^‐pentobarbital, via the left marginal ear vein) and removal of the heart, which was retrogradely perfused via the aorta for enzymatic dissociation of left atrial cells (Workman *et al*. 2000). Right atrial tissues were obtained from 28 adult patients (66 ± 2 years; 20 male, eight female) undergoing cardiac surgery (82% coronary artery bypass graft, 36% aortic valve replacement). All patients were in sinus rhythm on the day of surgery. One patient had a history of paroxysmal AF; none had chronic AF; 75% had angina, 59% had hypertension, 21% had myocardial infarction and 15% had diabetes. Left ventricular ejection fraction was 61 ± 2% (including two patients <45%). Cardiac drugs: β‐blocker (79%), angiotensin‐converting enzyme inhibitor/angiotensin receptor‐blocker (39%), Ca^2+^ channel‐blocker (CCB) (29%), statin (79%), nitrate (50%), nicorandil (14%) and eplerenone (4%). Cardiomyocytes were isolated by the ‘chunk’ method (Workman *et al*. 2001), stored (≤9 h, ∼20 °C) in cardioplaegic solution (mm): 70 KOH, 40 KCl, 50 L‐glutamic acid, 20 taurine, 20 KH_2_PO_4_, 3 MgCl_2_, 10 glucose, 10 Hepes and 0.5 EGTA (pH 7.2).

### Whole‐cell‐patch clamp

Cardiomyocytes were superfused (35–37 °C) with a physiological salt solution (mm): 140 NaCl, 4 KCl, 1.8 CaCl_2_, 1 MgCl_2_, 11 glucose and 10 Hepes (pH 7.4). Microelectrodes contained (mm): 130 K‐aspartate, 15 KCl, 10 NaCl, 1 MgCl_2_, 10 Hepes and 0.1 K_2_EGTA (pH 7.25). The resulting liquid–liquid junction potential (+9 mV; bath relative to pipette) was compensated for *a priori* (Neher, 1992). Microelectrode resistances were ∼3–6 MΩ. Associated voltage errors during peak *I*_CaL_ recording were minimized by bridge‐balancing prior to sealing and expected to be <5 mV; no subsequent series resistance compensation was applied. Membrane currents and APs were stimulated and recorded by whole‐cell‐patch clamp, with an AxoClamp 2B amplifier (Axon Instruments, Foster City, CA, USA) and WinWCP (J. Dempster, University of Strathclyde, Glasgow, UK) or Clampex (Axon Instruments) software. Ruptured (rather than perforated) patch configuration was chosen for its relative ease of controlling access resistance and because preservation of *I*_CaL_‐associated intracellular signalling was not required when dynamic clamping (when *I*_CaL_ was necessarily inhibited). *I*_CaL_ was stimulated with 300 ms voltage pulses (Fig. 2 *A*). Pulse frequency was 1 Hz, for best approximation of the influence of Ca^2+^‐induced inactivation of *I*_CaL_ in the model *I*_CaL_ when dynamic clamping APs at similar rates. Contaminating currents were suppressed by 4‐aminopyridine (4‐AP; 5 mm), niflumic acid (0.1 mm) and TTX (5 µm). APs were recorded under current clamp in bridge‐mode, stimulated continuously with pulses of 1–2 nA amplitude, 3–5 ms duration, 0.7‐1 Hz frequency, +/– a small holding current (<1.5 pA pF^–1^, to gain an initial resting *V*_m_ of ∼‐80 mV); parameters were kept constant throughout protocol. In some myocytes, [Ca^2+^]_i_ was measured concurrently with APs by epifluorescence microscopy, with ratiometric quantification of [Ca^2+^]_i_ using Fura‐2 pentapotassium salt, 0.1 mm (with K_2_EGTA then lowered to 0.01 mm) via the patch pipette. Fluorescence was measured at 340 and 380 nm every 15 ms using a Cairn Optoscan monochromator (Cairn Research, Faversham, UK). Minimum (*R*_min_) and maximum (*R*_max_) fluorescence ratios (340/380 nm) were measured; cytoplasmic [Ca^2+^] was calculated as *K*_d _× [(*R* – *R*_min_)/(*R*_max_ – *R*)], with *K*_d_ of 1.2 µm (Kettlewell *et al*. 2013).

### Dynamic clamp technique

**Figure 1:**
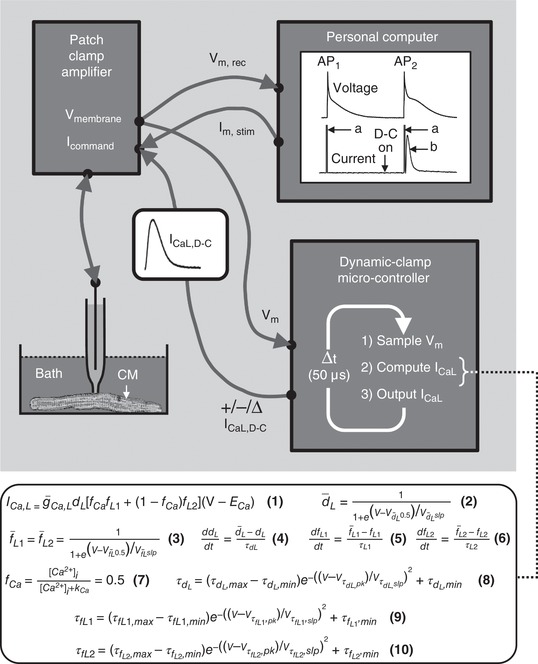
Dynamic clamp set‐up and method Personal computer (PC) running standard electrophysiology software to generate a train of short pulses (‘a’), which are sent (*I*_m, stim_) to input (*I*_command_) of a patch clamp amplifier (PCA) instructing it to inject current pulses to elicit action potentials (AP) in an isolated cardiomyocyte (CM). The membrane voltage (*V*_m_) change is measured by PCA and sent (*V*_m, rec_) from PCA output (*V*_membrane_) for display and recording on the PC (i.e. conventional AP recording) (‘AP_1_’). When the dynamic clamp (D‐C) microcontroller is switched on (‘D‐C on’), it continuously samples *V*_m_ (from PCA *V*_membrane_) during subsequent AP recording and, using Hodgkin–Huxley equations (eqns 1‐10; definitions in the Methods), calculates a model L‐type Ca^2+^ current (*I*_CaL_) based on instantaneous *V*_m_, which is updated and injected, as a dynamic clamp current (*I*_CaL,D‐C_), into the stimulus pathway (via *I*_command_) at precisely timed 50 µs intervals. Because *I*_CaL,D‐C_ injection modifies *V*_m_, and *vice versa*, there is a bidirectional dynamic interaction between the waveforms of the ensuing AP (‘AP_2_’) and corresponding *I*_CaL,D‐C_ (‘b’). Parameters in eqns (1), (2), (3), (4), (5), (6), (7), (8), (9), (10) are initially set with control values measured in atrial cells (Fig. 2), then systematically changed to investigate effects of altering magnitude, polarity, voltage‐ and/or time‐dependence of *I*_CaL,D‐C_ (‘+/‐/∆ *I*_CaL,D‐C_’) on AP_2_ and subsequent APs.

**Figure 2:**
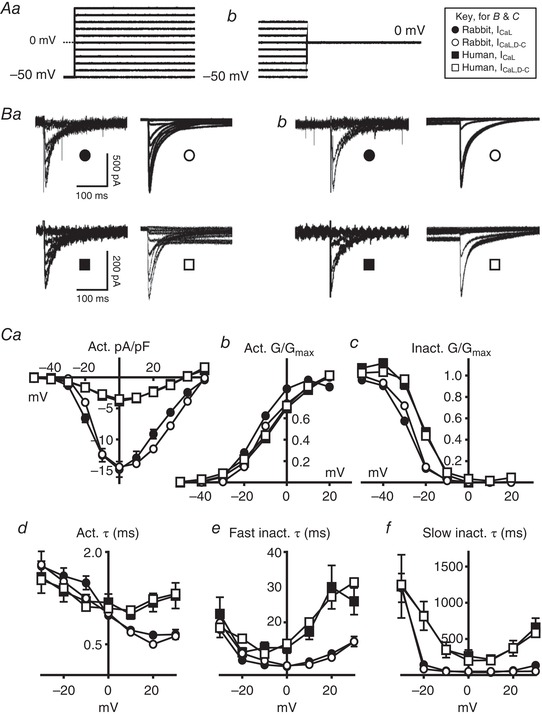
Comparison of live and simulated atrial *I*_CaL_ in rabbit and human Circles, rabbit; squares, human. Filled symbols, live atrial cell data (*I*_CaL_); open symbols, current generated by dynamic clamp *I*_CaL_ models (*I*_CaL,D‐C_). *A*, voltage protocols for steady‐state activation (*Aa*) and inactivation (*Ab*) of *I*_CaL_ or *I*_CaL,D‐C_, producing currents in (*Ba*) and (*Bb*). *C*, mean ± SE voltage‐ and time‐dependence of *I*_CaL_ activation and inactivation in live cells (*n* = 22–35 cells/four rabbits; 13–43 cells/seven patients), or *I*_CaL,D‐C_ from single simulation runs. *Ca*, steady‐state activation current density. *Cb* and *Cc*, steady‐state activation and inactivation conductance (G). *Cd*, activation time constant. *Ce* and *Cf*, fast and slow inactivation time constants.

The dynamic clamp technique (Fig. 1) was used, during AP recording in rabbit and human atrial myocytes, to simulate systematic changes in the voltage‐dependent characteristics of steady‐state activation and inactivation of *I*_CaL_. Native *I*_CaL_ was first inhibited with nifedipine (3 µm), then replaced by a mathematically modelled, dynamic clamp current, *I*_CaL,D‐C_. This current was computed in real‐time, based on the myocyte's continuously monitored *V*_m_ during the AP, and injected into the cell via the patch‐pipette (by adding it to the AxoClamp 2B stimulus current pathway) at precisely‐timed 50 µs intervals (update rate: 20 kHz). Because the *I*_CaL,D‐C_ injection in turn modifies the *V*_m_, there is a bidirectional dynamic interaction between the AP waveform and *I*_CaL,D‐C_ (Fig. 1). The *I*_CaL_ model implemented in the dynamic clamp is a simplified version of that developed by Nygren *et al*. (1998) and exhibits exponential voltage‐dependent activation, as well as bi‐exponential inactivation with a fast component governed by [Ca^2+^]_i_. Equations (1), (2), (3), (4), (5), (6), (7), (8), (9), (10) (shown in Fig. 1 in general form, and also below containing values obtained from the experiments) define *I*_CaL_, governed by a voltage‐dependent activation factor ($d_{L}$) and exhibiting a bi‐exponential decay ($f_{L1},\;f_{L2}$), with semi‐empirical, steady‐state activation (${\bar{d}}_{L}$), inactivation (${\bar{f}}_{L}$) and time constant curves (τ_d_,τ_fL1_,τ_fL2_); all tuned to the voltage‐ and time‐dependent parameters of the native *I*_CaL_ (Fig. 2). In the absence of information on [Ca^2+^]_i_ accessible to the dynamic clamp, the Ca^2+^‐dependency of *I*_CaL_ inactivation was simplified by fixing *f*_Ca_ (determining relative proportion of fast and slow decay) at 0.5. The dynamic clamp was custom‐built using a National Instruments CRIO‐9076 real time controller, equipped with a 400 MHz PowerPC CPU, Xilinx Spartan 6 FPGA (Field Programmable Gate Array) and 16 bit A/D and D/A converters (National Instruments, Newbury, UK). The *I*_CaL_ model, executing on the FPGA, was written in LabVIEW (National Instruments) and the device controlled from a host computer via an RS232 interface. The user interface, for defining and controlling *I*_CaL,D‐C_ characteristics, was custom written: WinWCP DCLAMP (John Dempster). By systematically and independently shifting half‐voltages (*V*_0.5_) of *I*_CaL,D‐C_ activation and inactivation, via this interface, the effects of changing the *I*_CaL,D‐C_ window region on AP characteristics including EADs could be investigated. All effects were checked for reversibility upon removal of such *I*_CaL,D‐C_ interventions.

### *I*_CaL_ models implemented in the dynamic clamp, containing values from the experimental data shown in Fig. 2

Rabbit:

(1) I Ca ,L=g¯ Ca ,LdLf Ca fL1+1−f Ca fL2V−49.5

(2) $${\bar{d}}_{L}=\frac{1}{1+e^{\left(V+14.6\right)/4.5}}$$

(3) $${\bar{f}}_{L1}={\bar{f}}_{L2}=\frac{1}{1+e^{\left(V+28.3\right)/4.4}}$$

(4) ddLdt=d¯L−dLτ dL

(5) $$\frac{df_{L1}}{dt}=\frac{{\bar{f}}_{L1}-f_{L1}}{\tau_{L1}}$$

(6) $$\frac{df_{L2}}{dt}=\frac{{\bar{f}}_{L2}-f_{L2}}{\tau_{L2}}$$

(7) f Ca =Ca2+iCa2+i+k Ca =0.5

(8) τ dL =0.0018e−V+21/252+0.0024

(9) τ fL 1=0.74e−V+29.6/5.82+0.066

(10) τ fL 2=29.49e−V−1/18682+29.5

Human:

(1) I Ca ,L=g¯ Ca ,LdLf Ca fL1+1−f Ca fL2V−38.4

(2) $$\;{\bar{\;d}}_{L}=\frac{1}{1+e^{\left(V+6.5\right)/7.8}}$$

(3) $${\bar{f}}_{L1}={\bar{f}}_{L2}=\frac{1}{1+e^{\left(V+23\right)/3.8}}$$

(4) ddLdt=d¯L−dLτ dL

(5) $$\frac{df_{L1}}{dt}=\frac{{\bar{f}}_{L1}-f_{L1}}{\tau_{L1}}$$

(6) $$\frac{df_{L2}}{dt}=\frac{{\bar{f}}_{L2}-f_{L2}}{\tau_{L2}}$$

(7) f Ca =Ca2+iCa2+i+k Ca =0.5

(8) τ dL =0.008e−V+7.5/52.32+0.0023

(9) τ fL 1=6.01e−V−6.3/88.32+6.21

(10) τ fL 2=0.019e−V+9.2/22.62+0.023

### Strengths and weaknesses of the technique

A significant strength of the dynamic clamp technique is that, by simulating modification of a specific ion current in a real cell (rather than in a mathematical model), its effect on the AP waveform can be observed interacting with all the naturally occurring voltage‐ and time‐dependent physiological processes, including those not yet discovered and incorporated into the models. This is not to say that dynamic clamping is without its own intrinsic limitations, with the main ones being: (i) Because the modelled ion channel current is injected through the patch pipette and is thus carried primarily by ions such as K^+^, Cl^−^, or aspartate, the ‘chemical effects’ of the specific ion current modelled (e.g. *I*_CaL_), such as local increases in ion concentrations (e.g. Ca^2+^) or signalling changes from conformational changes of the channel, are absent (Berecki *et al*. 2014). However, this can also be an opportunity to separate electrical effects from chemical effects of the current (Berecki *et al*. 2014), as in the present study. (ii) The model current (in this case, *I*_CaL,D‐C_) is based on voltage‐ and time‐dependent parameters averaged from recordings made from multiple cells rather than from the individual cell being dynamic clamped. (iii) The dynamic clamp technique naturally shares some of the limitations of conventional voltage‐ and current clamp methods, such as the use of fixed intracellular and extracellular solutions, and artefacts of electrode resistance and capacitance (Prinz *et al*. 2004).

### Statistical analysis

Electrophysiological and [Ca^2+^]_i_ data are expressed as the mean ± SE. Continuous data were compared, among three or more groups, using a Friedman repeated measures test, for non‐parametric, matched data, followed by Dunn's multiple comparisons test. Unpaired data were compared using a Kruskal–Wallis test, followed by uncorrected Dunn's. Categorical data were compared using a chi‐squared test with Yates’ correction. *P* < 0.05 was considered statistically significant. All statistical and curve fitting analyses were performed using Prism, version 7.00 (GraphPad Software Inc., San Diego, CA, USA).

## Results

### Comparison of live and simulated atrial *I*_CaL_ in rabbit and human

L‐type Ca^2+^ current (*I*_CaL_) showed characteristic rapid, mono‐exponential activation, and slow, bi‐exponential decay, in rabbit and human atrial myocytes (Fig. 2 *B* and *C*). Peak *I*_CaL_ density was 3.9‐fold larger in rabbit than human (Fig. 2 *Ca*), with corresponding *G*_max_ values of 15 nS (0.3 nS pF^–1^) and 7 nS (0.1 nS pF^–1^), respectively, but the voltage‐dependence of steady‐state activation was similar (Fig. 2 *Cb*). Other voltage‐ and time‐dependent *I*_CaL_ characteristics differed between species, mainly with human having a positively‐shifted steady‐state inactivation compared to rabbit (Fig. 2 *Cc*), and also slower activation (Fig. 2 *Cd*) and inactivation (Fig. 2 *Ce* and *Cf*) at positive potentials. Dynamic clamp currents (*I*_CaL,D‐C_), injected into a simple resistor (500 MΩ) capacitor (33 pF) circuit (Patch‐1U model cell; Axon Instruments) and simulated in response to the same voltage pulse protocols (Fig. 2 *Aa* and *Ab*), showed generally close agreement with the live cell data for all voltage‐ and time‐dependent parameters (Fig. 2 *B* and *C*).

### Validation of the dynamic clamp technique for *I*_CaL_

**Figure 3:**
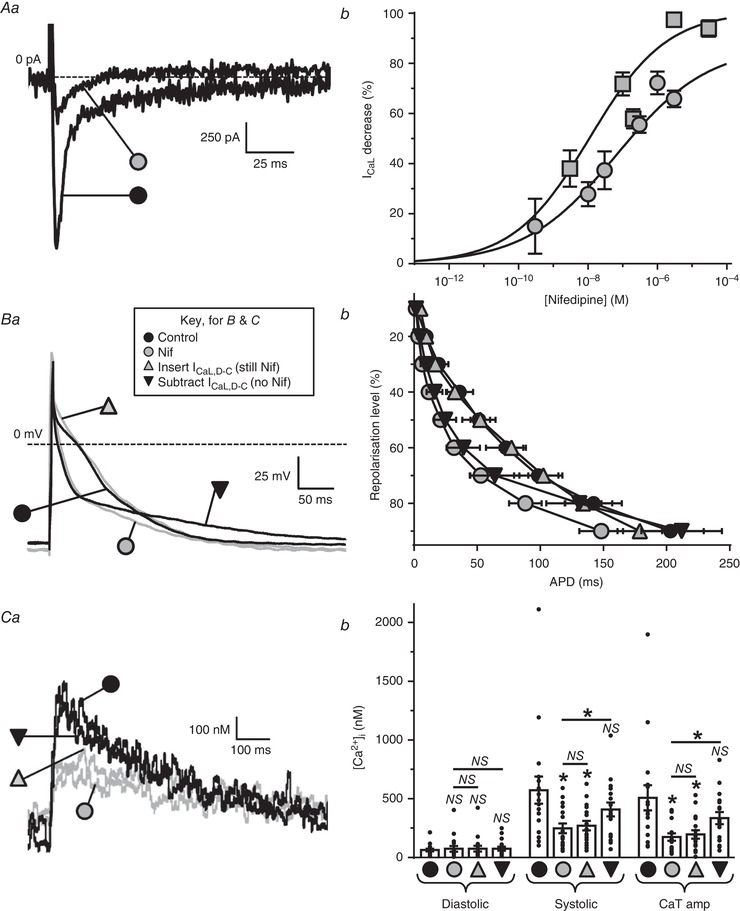
Validation of dynamic clamp technique for *I*_CaL_ in rabbit atrial cells Grey symbols = nifedipine (Nif) present; black = Nif absent. *Aa*, original current traces showing typical maximal *I*_CaL_‐decrease by Nif (3 µm) in a rabbit atrial cell. *Ab*, Nif‐*I*_CaL_ concentration–response curve for rabbit cells (*n* = 5–14, 3 or 4 rabbits; circles); with human cells (*n* = 10–22 cells/3 patients; squares) for comparison. *Ba*, representative superimposed APs from a single atrial cell before (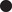) and after (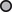) 3 µm Nif, during insertion of *I*_CaL,D‐C_ (*G*_max_ 15 nS) in the continued presence of Nif (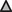), and during subtraction of *I*_CaL,D‐C_ (*G*_max_ –15 nS) in the absence of Nif (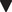). *Bb*, average AP duration (APD) in 19–22 cells/8 or 9 rabbits under the same conditions as (*Ba*). *Ca*, [Ca^2+^]_i_ transients (CaT) measured during AP recording under the same conditions as (*B*). *Cb*, average [Ca^2+^]_i_ in 17 cells/6 rabbits from (*Bb*). ^*^ *P* < 0.05 *vs*. controls, or between groups under lines; NS, not significant; Friedman & Dunn's multiple comparisons tests.

Native *I*_CaL_ was substantially inhibited, as shown in a rabbit atrial myocyte, using nifedipine (Fig. 3 *Aa*). Nifedipine concentration––response curves (Fig. 3 *Ab*) indicated complete inhibition of human atrial *I*_CaL_ (*I*_max_ 101%), but only partial inhibition in rabbit (*I*_max_: 87%). Nifedipine at 3 µm, chosen as the highest concentration not substantially inhibiting currents other than *I*_CaL_ (e.g. *I*_Kur_ or *I*_TO_) (Gao *et al*. 2005) was used in both species to allow dynamic clamp insertion of *I*_CaL,D‐C_ during AP recording. This inhibited native *I*_CaL_ by 91% in human and 68% in rabbit (Fig. 3 *Ab*). In rabbit atrial cells, nifedipine removed a substantial portion of the AP plateau (Fig. 3 *Ba*), with a corresponding marked decrease in APD_50–90_ (Fig. 3 *Bb*), and also markedly decreased Ca^2+^ transient amplitude, CaT (Fig. 3 *Ca* and *Cb*). Subsequent insertion of *I*_CaL,D‐C_, still with nifedipine superfusion, fully restored the plateau and all but the latest phases of repolarization (∼APD_90_) (Fig. 3 *Bb*), and without further effect on CaT (Fig. 3 *Ca* and *Cb*). Without nifedipine, subtraction of *I*_CaL,D‐C_, which offset the electrical (but not [Ca^2+^]_i_) effects of *I*_CaL_, did not alter [Ca^2+^]_i_ (Fig. 3 *Ca* and *Cb*) and produced similar APD_50‐70_ changes as nifedipine, although it left a clear residual ‘AP foot’ (prolonged APD_80‐90_ in Fig. 3 *Ba* and *Bb*).

### Mathematical modelling of the contribution to AP of electrical *vs*. Ca^2+^ changes from *I*_CaL_

**Figure 4:**
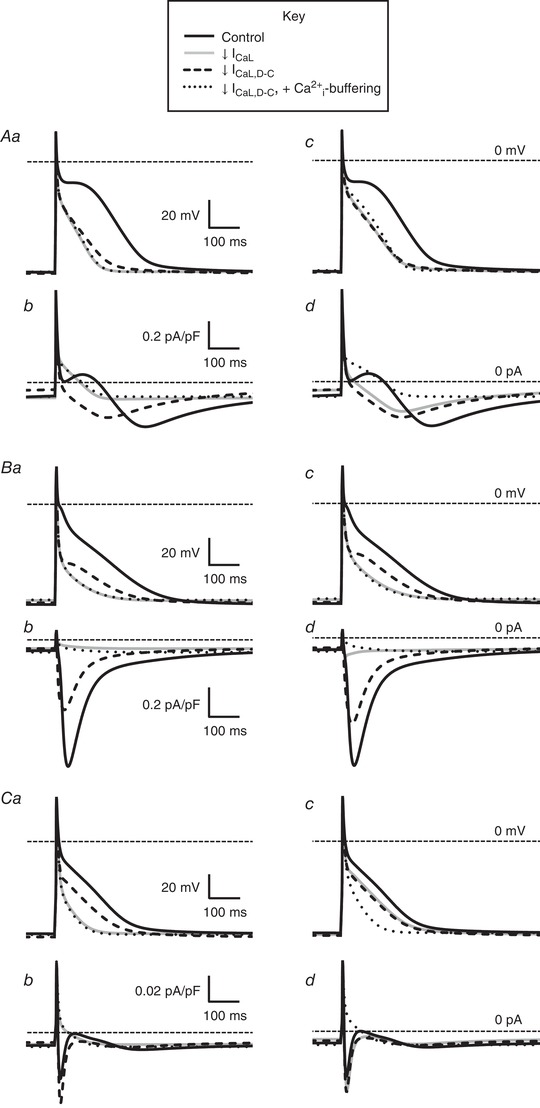
Mathematical modelling of the contribution to the AP waveform of electrical and Ca^2+^ changes from *I*_CaL_ decrease, and the involvement of *I*_Na/Ca_ Action potentials (AP) and Na^+^/Ca^2+^‐exchange currents (*I*_Na/Ca_) simulated using mathematical models CRN (Courtemanche *et al*. 1998) (*A*), Grandi (Grandi *et al*. 2011) (*B*) and Nygren (Nygren *et al*. 1998) (*C*). *Aa*, *Ab*, *Ba*, *Bb*, *Ca* and *Cb*, superimposed APs and *I*_Na/Ca_, respectively (1 Hz stimulation), under a 100% reduction in *I*_CaL_ or dynamic clamp *I*_CaL_ (*I*_CaL,D‐C_). *Ac*, *Ad*, *Bc*, *Bd*, *Cc* and *Cd* are APs and *I*_Na/Ca_ under a 70% reduction in *I*_CaL_ or *I*_CaL,D‐C_. Solid black lines indicate the control; solid grey lines indicate *I*_CaL_ reduction (e.g. as from nifedipine); dashed black lines indicate *I*_CaL,D‐C_ reduction (i.e. isolated electrical component of *I*_CaL_ reduction); dotted black lines indicate *I*_CaL,D‐C_ reduction of 100% (*Aa*, *Ab*, *Ba*, *Bb*, *Ca* and *Cb*) or 70% (*Ac*, *Ad*, *Bc*, *Bd*, *Cc* and *Cd*) when holding [Ca^2+^]_i_ at its diastolic level (100% Ca^2+^ buffering).

The relative contributions of purely electrical (*I*_CaL,D‐C_) *vs*. ‘chemical’ (Ca^2+^) effects of *I*_CaL_‐reduction, as well as the likely cause of the ‘AP foot’ in the above (*I*_CaL,D‐C_ subtraction without nifedipine) experiment, were investigated using three published mathematical models of human atrial APs: ‘CRN’ (Courtemanche *et al*. 1998), ‘Grandi’ (Grandi *et al*. 2011) and ‘Nygren’(Nygren *et al*. 1998) (Fig. 4). In each model, abolition of *I*_CaL_ (and thus Ca^2+^‐induced Ca^2+^‐release, CICR) abolished both the AP plateau (panels *Aa*, *Ba* and *Ca*) and inward *I*_Na/Ca_ (panels *Ab*, *Bb* and *Cb*). However, compared to *I*_CaL,D‐C_‐abolition in the presence of full [Ca^2+^]_i_‐buffering (also abolishing CICR and inward *I*_Na/Ca_), *I*_CaL,D‐C_‐abolition alone only suppressed the plateau, with the difference (i.e. between dotted and dashed line pairs) being a result of inward *I*_Na/Ca_. Qualitatively similar effects of 70% *I*_CaL_‐ or *I*_CaL,D‐C_‐reduction on APs (panels *Ac*, *Bc* and *Cc*) and *I*_Na/Ca_ (panels *Ad*, *Bd* and *Cd*) were observed, although generally attenuated compared to the 100% reductions, with ‘Grandi’ best qualitatively simulating (Fig. 4 *Bc*) the ‘AP foot’ seen in rabbit atrial cells, accompanied by a relatively large inward *I*_Na/Ca_.

### Effects of dynamic clamp‐widening of the *I*_CaL_ window region on atrial AP waveforms

**Figure 5:**
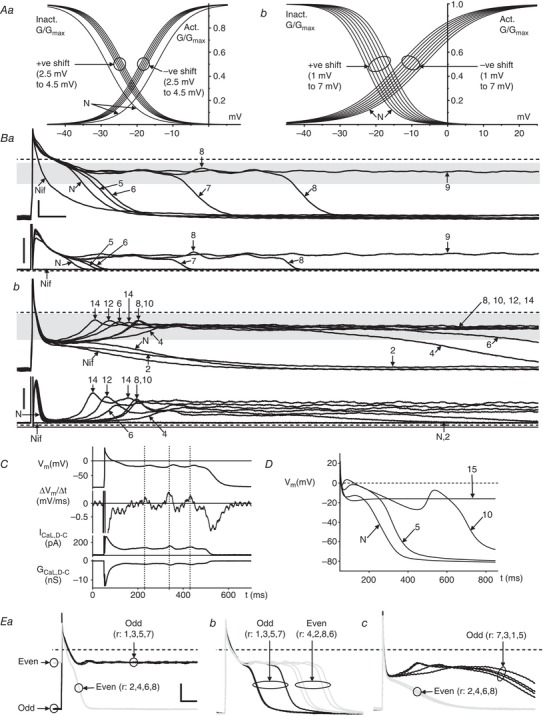
Effects of dynamic clamp‐widening of the *I*_CaL_ window on APs in rabbit and human *A*, *I*_CaL,D‐C_‐voltage curves used to progressively widen window region during recording of APs (*B*) by simultaneous stepwise shifting of half‐voltages (*V*_0.5_) of activation (negatively) and inactivation (positively) from normal (N), in rabbit (*Aa*) (widening by 5–9 mV in 1 mV steps, by 0.5 mV shifting of activation and inactivation) and human (*Ab*) (2–14 mV in 2 mV steps). *B*, superimposed APs (upper) and *I*_CaL,D‐C_ (lower) from a single rabbit (*Ba*) and human (*Bb*) atrial cell, recorded first with 3 µm nifedipine (Nif), then after inserting *I*_CaL,D‐C_ (N), followed by window widening by arrowed values (mV). AP responses to widening include EADs, and instances of non‐repolarization; *I_CaL_*_,D‐C_ responses: associated reactivations, and non‐inactivations. All traces at same scale (bars = 50 ms, 25 mV). Grey bands = window voltage ranges, as determined from (*Aa*) and (*Ab*), producing EADs. *I*_CaL,D‐C_ bar = 0.2 nA for (*Ba*), 0.1 nA for (*Bb*). *C*, temporal relationship of changes in *V*_m_ (top trace; from *Ba*, 8 mV widening), its first derivative (Δ*V*_m_/Δ*t*), and D‐C current (*I*_CaL,D‐C_) and conductance (G_CaL,D‐C_). *D*, mathematical simulation, using CRN (Courtemanche *et al*. 1998), of AP responses to widening *I*_CaL_ window by 5, 10 and 15 mV beyond N, showing production of APD‐increase, EAD and non‐repolarizing AP. *E*, sets of eight superimposed consecutive AP recordings (*r*: 1–8; black trace = odd *r* number, grey = even) from rabbit (*Ea* and *Eb*) and human (*Ec*), showing bi‐alternation of MDP (*Ea*) or APD (*Eb* and *Ec*) after 10 mV window widening. Bars = 100 ms, 25 mV.

**Figure 6:**
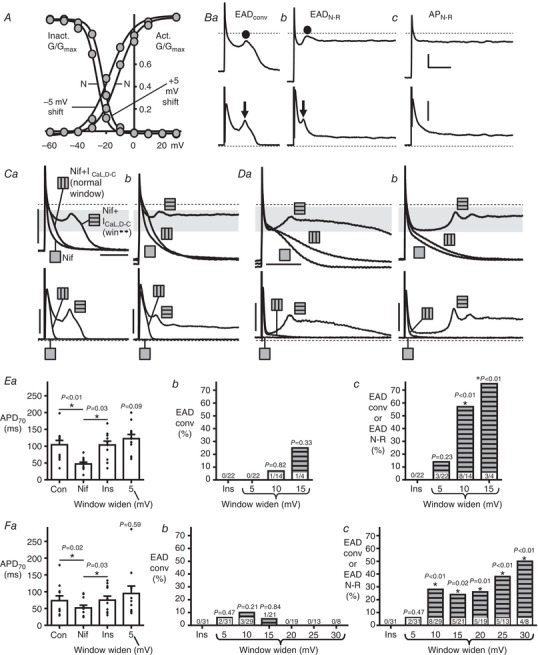
Symmetrical widening of the *I*_CaL,D‐C_ window produces EADs in rabbit and human atrial cells *A*, *I*_CaL,D‐C_‐voltage curves to widen window region during AP recording; in this case by 10 mV, in rabbit atrial, by simultaneously shifting activation *V*_0.5_ –ve by 5 mV (*vs*. normal, N) and inactivation *V*_0.5_ +ve 5 mV. *B*, EAD categorization for (*C*) to (*F*). *Ba*, EAD_conv_ = conventional EAD: clear transient depolarization in AP phase 2–3 followed by normal repolarization; *Bb*, EAD_N‐R_ = EAD followed by non‐repolarizing (within BCL) AP; *Bc*, AP_N‐R _= non‐repolarizing AP without EAD. 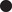  = EAD. ↓ = *I*_CaL,DC_ reactivation. All traces (rabbit, 10 mV widening) at same scale: bar on AP = 25 mV; *I*_CaL,D‐C_ = 0.2 nA; time = 100 ms. APs (upper) and *I*_CaL,D‐C_ (lower) from (*C*) rabbit and (*D*) human, with 3 µM Nif (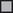), insertion of *I*_CaL,D‐C_ with normal‐window (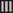), 10 mV widening (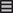), showing production of EAD_conv_ (*Ca* and *Da*) and EAD_N‐R_ (*Cb* and *Db*). Bars = 50 mV for APs; 0.2 nA for rabbit *I*_CaL,D‐C_, 0.1 nA for human; 100 ms for rabbit; 200 ms for human. Grey bands = window voltage ranges, from *I*_CaL,D‐C_‐voltage curves, producing EADs. Average AP and EAD from rabbit (*E*) and human (*F*). *Ea* and *Fa*, APD_70_ (for APs without EADs) in control (Con), following Nif, then *I*_CaL,D‐C_‐insertion (Ins) and 5 mV widening; *n *= 12 cells/6 rabbits, 11 cells/8 patients. Incidences of cells displaying EAD_conv_ (*Eb* and *Fb*) or EAD_conv_/EAD_N‐R_ (*Ec* and *Fc*) after *I*_CaL,D‐C_‐insertion and progressive window widening (5–15 mV in rabbit; 5‐30 mV human). Values within bars: cell *n* (3–6 rabbits/4–8 patients). *P*: compares groups under horizontal lines, or *vs*. ‘Ins’. Friedmans, Dunn's multiple comparisons, chi‐squared tests.

The overlapping region of the respective activation and inactivation curves ‘N’ in Fig. 5 *A* (taken from Fig. 2 *Cb* and *Cc*) signifies the normal *I*_CaL,D‐C_ window region. This is the *V*_m_ range within which a fraction of the current not inactivated may be available for reactivation. During continuous AP recording, insertion of normal‐window *I*_CaL,D‐C_ resulted in a normal AP waveform (‘N’) in each of six rabbit and six human atrial myocytes (Fig. 5 *Ba* and *Bb*, upper), with the corresponding *I*_CaL,D‐C_ ‘N’ shown beneath. Note that *I*_CaL,D‐C_ has opposite polarity to conventionally recorded *I*_CaL_ because it is injected into the cell. However, when the window region was progressively widened, in small (1 mV) steps, by simultaneously and equally (i.e. symmetrically) shifting half‐voltages (*V*_0.5_) of activation and inactivation (Fig. 5 *A*), a variety of changes in AP waveforms was observed. In all 12 myocytes, initial window widening increased APD, accompanied by slowing of *I*_CaL,D‐C_ decay. As the window width was increased further, EADs were produced, either singly or with one or more subsequent oscillations, accompanied by *I*_CaL,D‐C_ reactivation (Fig. 5 *B*). The take‐off *V*_m_ of each single (or 1st‐of‐a‐train) EAD ranged −22 mV to −16 mV in rabbit and −44 mV to −21 mV in human. The maximum *V*_m_ of this EAD or any subsequent oscillation(s) was −5 mV in rabbit and −8 mV in human; minimum *V*_m_ of any subsequent oscillation(s): −22 mV in rabbit, −28 mV in human. The maximum rate of rise of each EAD upstroke coincided with the peak of the corresponding reactivating current (*I*_CaL,D‐C_) and conductance (G_CaL,D‐C_) (Fig. 5 *C*). In one of the rabbit atrial myocytes (Fig. 5 *Ba*), initial window widening produced exclusively EADs that were followed by full repolarization (i.e. conventional EADs) then, with further widening, exclusively EADs on APs failing to repolarize (within BCL). In two of the rabbit myocytes, both EAD types were observed at same window widths, as it was progressively widened, and, in the other three, only EADs preceding non‐repolarization occurred. Of the six human atrial myocytes, two showed a progressive shift from predominantly conventional EADs to predominantly EADs with non‐repolarization (Fig. 5 *Bb*), with four showing exclusively EADs with non‐repolarization. All effects of window widening were reversed upon returning to normal‐window width *I*_CaL,D‐C_ at the end of each protocol. Simulation of stepwise widening of the *I*_CaL_ window, using ‘CRN’ (Courtemanche *et al*. 1998), also produced progressive APD‐increase, conventional EADs and, finally, repolarization failure (Fig. 5 *D*). A further observation in the live cells, albeit uncommon, was a consistently repeating bi‐alternation of AP‐waveform: alternans (Fig. 5 *E*). This occurred in three of 22 rabbit and two of 31 human atrial cells (includes cells from Fig. 6 *E* and *F*), and featured both maximum diastolic potential (MDP)‐alternans (Fig. 5 *Ea*) and true APD‐alternans (i.e. with no accompanying MDP‐alternans) (Fig. 5 *Eb* and *Ec*).

### *I*_CaL,D‐C_ window width‐dependence of EAD incidence in rabbit and human atrium

A systematic study of the effect of symmetrical widening of the *I*_CaL,D‐C_ window (Fig. 6 *A*) on the incidence of the two different EAD types (as defined in Fig. 6 *Ba* and *Bb*) showed a window width‐dependence of EAD production in rabbit and human atrial myocytes. Note that the AP response‐type in Fig. 6 *Bc* displayed no EAD and so was not included in the analyses. Representative traces in Fig. 6 *C* (rabbit) and *D* (human) illustrate the respective average data (Fig. 6 *E* and *F*), showing that, after insertion of normal‐window width *I*_CaL,D‐C_ to recover the AP plateau pre‐supressed by nifedipine (Fig. 6 *Ea* and *Fa*), increasing the window width (in 5 mV steps up to 15 mV in rabbit, 30 mV in human) produced a significant, stepwise, increase in the incidence of combined‐type EADs (Fig. 6 *Ec*: rabbit; Fig. 6 *Fc*: human), with no significant effect on the incidence of solely conventional EADs (Fig. 6 *Eb* and *Fb*).

### Asymmetrical widening of the *I*_CaL,D‐C_ window produced EADs, particularly when shifting activation voltage

**Figure 7:**
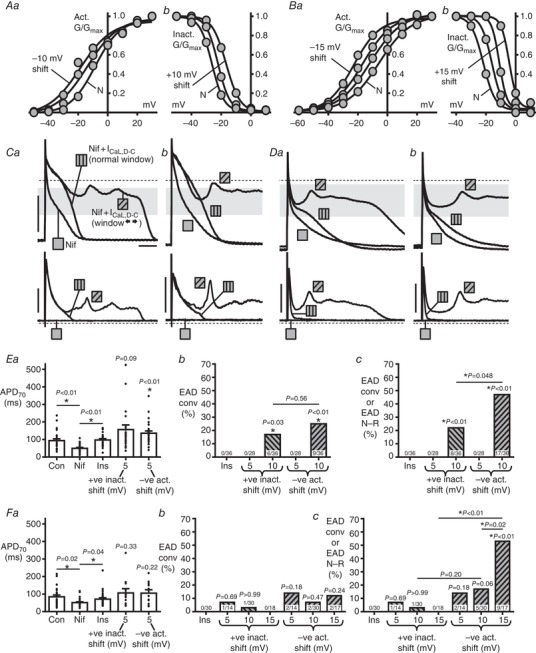
Asymmetrical widening of *I*_CaL,D‐C_ window, particularly by shifting activation voltage, produces EADs *I*_CaL,D‐C_‐voltage curves used for rabbit (*A*) and human (*B*), showing *V*_0.5_ shifts of (*Aa* and *Ba*) activation and (*Ab* and *Bb*) inactivation (5 mV steps *vs*. N) to asymmetrically widen the *I*_CaL,D‐C_‐window during AP recording. APs (upper) and *I*_CaL,D‐C_ (lower) from (*C*) rabbit and (*D*) human, with 3 µm Nif (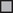), insertion of *I*_CaL,D‐C_ with normal‐window (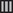) then –10 mV activation shift (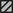), showing EAD_conv_ (*Ca* and *Da*) and EAD_N‐R_ (*Cb* and *Db*). Bars (all traces) = 50 mV, 0.1 nA, 100 ms. Grey bands = window voltage ranges, as determined from (*A*) and (*B*), producing EADs. Average AP and EAD from rabbit (*E*) and human (*F*). *Ea* and *Fa*, APD_70_ in control (Con), following Nif, *I*_CaL,D‐C_‐insertion (Ins) and a subsequent +5 mV shift in *V*_0.5_ inactivation or, separately, a –5 mV activation shift (*n*=26 cells/9 rabbits, 11–28 cells/4–10 patients; Friedman or Kruskal–Wallis, Dunn's tests). Incidences of cells displaying EAD_conv_ (*Eb* and *Fb*) or EAD_conv_/EAD_N‐R_ (*Ec* and *Fc*) after *I*_CaL,D‐C_‐insertion and subsequent act/inactivation *V*_0.5_ shifting (5 mV steps). *n *= 9 or 10 rabbits/4–10 patients. *P*: compares groups under lines, or *vs*. Ins.

Widening *I*_CaL,D‐C_ window asymmetrically, by shifting independently (solely) either the activation or inactivation curves (e.g Fig. 7 *A* for rabbit; Fig. 7 *B* for human), also produced EADs, although preferentially from shifting activation. In rabbit, significant production of conventional EADs resulted from a 10 mV shift of either curve (Fig. 7 *Ca* and *Eb*). Considering combined‐type EADs (Fig. 7 *Ca* and *Cb*), a significantly higher incidence resulted from negatively‐shifting the activation curve compared to positively‐shifting inactivation (Fig. 7 *Ec*). Such a differential effect of activation *vs*. inactivation curve‐shifting was also significant in human (Fig. 7 *Da*, *Db* and *Fc*), and more pronounced than in rabbit, with no EADs resulting from a 15 mV shift in inactivation, yet with a 53% EAD incidence in response to an activation voltage‐shift of same magnitude (Fig. 7 *Fc*).

### Narrowing the *I*_CaL,D‐C_ window abolished EADs evoked by increasing current conductance with or without 4‐AP

**Figure 8:**
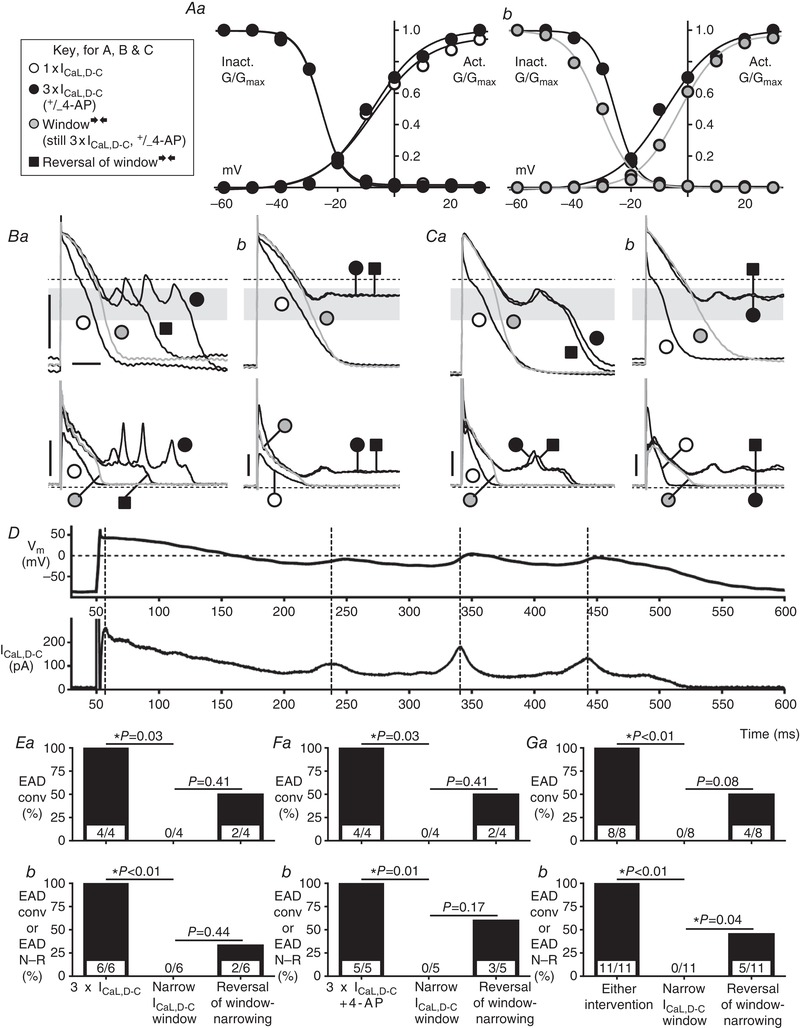
Narrowing *I*_CaL,D‐C_ window abolishes EADs from increased *I*_CaL,D‐C_ and/or 4‐AP in rabbit *A*, *I*_CaL,D‐C_‐voltage curves to narrow window region during AP recording. *Aa*, activation/inactivation curves, of normal window width, from either 1 × *I*_CaL,D‐C_ G_max_: 15 nS (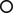) or 3 × G_max_: 45 nS (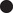); window unaltered by the *G*_max_‐increase. *Ab*, comparison of the normal‐width 45 nS curves (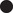) *vs*. 45 nS curves with window narrowed by simultaneously shifting *V*_0.5act_ +5 mV and V_0.5inact_ ‐5 mV (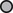). *B* and *C*, APs (upper) and *I*_CaL,D‐C_ (lower), all with Nif to supress *I*_CaL_. 15 nS *I*_CaL,D‐C_ with normal‐window (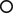), then EAD_conv_ (*Ba* and *Ca*) or EAD_N‐R_ (*Bb* and *Cb*) were evoked, either by trebling *I*_CaL,D‐C_ (*B*) or, in different cells, by trebling *I*_CaL,D‐C_ and adding 4‐aminopyridine (4‐AP; 2 mm) (*C*) (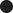). Then, the *I*_CaL,D‐C_ window was narrowed (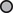), still with *G*_max_ 45 nS (and 4‐AP if applicable). Finally, window narrowing was reversed (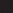). Bars = 50 mV, 0.1 nA, 100 ms. Grey bands = window voltage ranges, as determined from (*Aa*), producing EADs. *D*, timescale expansion of traces 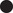 from (*Ba*), showing *I*_CaL,D‐C_–*V*_m_ temporal relationship during EAD‐formation. *E*–*G*, effect of window narrowing (and its reversal) on incidences of cells displaying EAD_conv_ (*Ea*, *Fa* and *Ga*), or EAD_conv_/EAD_N‐R_ (*Eb*, *Fb* and *Gb*), evoked by 3 × *I*_CaL,D‐C_ (*E*) or, in different cells, 3 × *I*_CaL,D‐C _+ 4‐AP (*F*), or either intervention (*G*). *n *= 2 rabbits in (*E*), *n *= 3 rabbits in (*F*), *n *= 5 rabbits in (*G*).

In rabbit atrial myocytes (*n *= 18), increasing (by three‐fold) the maximum conductance (G_max_) of normal‐window width *I*_CaL,D‐C_, which left the window region configuration entirely unaffected (Fig. 8 *Aa*), produced EADs in six myocytes, with conventional‐type in four, including multiple EADs (Fig. 8 *Ba*). The time scale‐expanded traces of Fig. 8 *D* shows these *V*_m_ oscillations and associated *I*_CaL,D‐C_ reactivations, along with the same temporal relationship between peak *I*_CaL,D‐C_ and EAD upstroke as observed in the earlier (Fig. 5 *C*) window widening experiments. In every cell, narrowing of the *I*_CaL,D‐C_ window (still at three‐fold *G*_max_) by 10 mV, by simultaneously and equally shifting *V*_0.5_ activation (positively) and inactivation (negatively) (Fig. 8 *Ab*), abolished all EADs of any type (Fig. 8 *B* and *E*). This effect was at least partially reversible (e.g. Fig. 8 *B*). In 10 of the 12 cells in which increasing *I*_CaL,D‐C_ alone did not produce EADs, 4‐AP was subsequently superfused, which, in turn, produced EADs (Fig. 8 *C*) in five cells. In each of those, the same window narrowing intervention, in the continued presence of increased *I*_CaL,D‐C_+4‐AP, also abolished all EADs of any type (Fig. 8 *C* and *F*). Combining the data (*n *= 11 myocytes) from either intervention affirmed the marked anti‐EAD effect of narrowing the *I*_CaL,D‐C_ window region (Fig. 8 *G*).

## Discussion

We validated the dynamic clamp technique for investigating, for the first time to our knowledge, the electrical contribution of *I*_CaL_ to atrial APs, and then made the following key findings: widening the ‘window’ *I*_CaL_ region, particularly asymmetrically by shifting voltage‐dependent *I*_CaL_ activation, produced EADs in a window width‐dependent manner in rabbit and human atrial myocytes, with some instances of AP‐alternans; and narrowing the *I*_CaL_ window abolished EADs that were produced by increasing *I*_CaL_ conductance and APD.

By informing and tuning the mathematical models of *I*_CaL_ used for dynamically injecting *I*_CaL,D‐C_, with the steady‐state voltage‐ and time‐dependent parameters of native *I*_CaL_ measured in the same cell types and under the same conditions as for dynamic clamping APs, this ensured an accurate and realistic representation of the native *I*_CaL_ during dynamic clamping APs. Some studies have used action potential clamping as a refinement of this step (e.g. for ventricular *I*_Kr_) (Altomare *et al*. 2015). Because native *I*_CaL_ must be pharmacologically inhibited in order to replace it with *I*_CaL,D‐C_ for systematic study of manipulating the *I*_CaL,D‐C_ window and, because dynamic clamp cannot inject Ca^2+^, there is no Ca^2+^‐induced Ca^2+^ release (CICR) during each AP. Therefore, a generalized (fixed) influence of Ca^2+^‐dependent inactivation of *I*_CaL_ (CDI) was included in *I*_CaL,D‐C_, by recording native *I*_CaL_ with minimal [Ca^2+^]_i_‐buffering (low [EGTA] to buffer reagent‐contaminating Ca^2+^ to ∼physiological diastolic [Ca^2+^]_i_) (Fig. 3 *C*) to permit the CaT during the initial voltage clamp experiments to affect the *I*_CaL_ waveform used to inform the model. *I*_CaL,D‐C_ did not, therefore, include a dynamically changing CDI. Other approaches to addressing CDI and absence of CICR when dynamic clamping have been to model subcellular [Ca^2+^] changes and feed back the influence of modelled CDI on the injected *I*_CaL,D‐C_ (Madhvani *et al*. 2011; Madhvani *et al*. 2015). Another consequence of CICR on the AP, *I*_Na/Ca_, has not been injected along with *I*_CaL,D‐C_ in any study to date. We inhibited native *I*_CaL_ using the best available CCB at the highest concentration possible without inhibiting important atrial repolarizing currents such as *I*_Kur_ and *I*_TO_ (i.e. nifedipine at 3 µM) (Gotoh *et al*. 1991; Gao *et al*. 2004; Gao *et al*. 2005). Whilst this substantially reduced *I*_CaL_ and CaT, it left a residual ∼30% *I*_CaL_ in rabbit atrial cells and ∼10% in human, which could be expected to produce a resulting component *I*_Na/Ca_. Nevertheless, following nifedipine application in rabbit atrial myocytes, which substantially suppressed AP plateau and shortened APD, we found that *I*_CaL,D‐C_‐insertion completely restored AP plateau and all but the latest phases of repolarization, thus validating *I*_CaL,D‐C_ for studying AP plateau and EADs. A recent simulation of ‘chemical’ *vs*. ‘electrical’ effects of *I*_Na/Ca_‐change on guinea‐pig ventricular APs also suggests only a small contribution from *I*_Na/Ca_ to late repolarization (Devenyi *et al*. 2017), although atrial APs were not studied. Conversely, in the present experiments, subtracting *I*_CaL,D‐C_ in the absence of nifedipine produced a residual ‘AP‐foot’, suggestive of inward *I*_Na/Ca_ under such a condition. Our mathematical modelling (with three AP models) (Courtemanche *et al*. 1998; Nygren *et al*. 1998; Grandi *et al*. 2011) of simultaneous APs and *I*_Na/Ca_, with and without full [Ca^2+^]_i_‐buffering, supported [Ca^2+^]_i_‐induced *I*_Na/Ca_ as the cause of this AP‐foot under *I*_CaL,D‐C_ subtraction. Dynamic clamp‐subtraction of ion currents has been used previously to attempt to overcome non‐specificity of a pharmacological blocker (Workman *et al*. 2012) or to convert between AP waveform types (e.g. changing a mouse ventricular AP to a more human‐like one using a calculated compensatory current) (Ahrens‐Nicklas & Christini, 2009). Whilst dynamic clamp‐subtraction of a suitable compensatory current (taking into account *I*_CaL_ and perhaps also *I*_Na/Ca_) could be conceived as an alternative to using nifedipine (before superimposing additional *I*_CaL_ conductances with altered window characteristics), implementation and interpretation would be highly challenging and it was not attempted in the present study.

A variety of AP, EAD, alternans, and *I*_CaL,D‐C_ responses resulted from widening *I*_CaL,D‐C_ window; each consistent with a major contribution from window *I*_CaL,D‐C_ reactivation. The EADs were always accompanied by *I*_CaL,D‐C_‐reactivation, and the coincidence of the *I*_CaL,D‐C_ and G_CaL,D‐C_ peak with the EAD upstroke maximum rate of rise is consistent with *I*_CaL,D‐C_ driving the EAD. The two main conditions for EAD production by window *I*_CaL_ reactivation (January & Riddle, 1989; Fozzard, 1992) were met, namely APD‐increase (allowing *I*_CaL,D‐C_ to dwell in the window region long enough to reactivate) and the EADs oscillating within a voltage range allowing *I*_CaL,D‐C_‐activation. For a contribution to these EADs from any inward *I*_Na/Ca_ (from any residual CICR), EAD take‐off potential would need to be negative to E_Na/Ca_, which is expected to be positive to ∼ −30 to −35 mV (Bers, 2002). Because the EADs in rabbit atrial myocytes (in which any residual CICR would be the larger) arose from potentials positive to E_Na/Ca_, this suggests they were caused mainly or solely by window *I*_CaL,D‐C_ reactivation. Some of the human atrial EADs arose from potentials negative to E_Na/Ca_, and so a potential contribution from inward *I*_Na/Ca_ to their upstroke cannot be excluded. However, any such contribution may be expected to be relatively small because native *I*_CaL_ was >90% inhibited. Furthermore, because *I*_Na/Ca_ decreases with depolarization, it cannot act regeneratively to enhance EAD amplitude unless [Ca^2+^]_i_ increases to maintain its driving force (Qu *et al*. 2013); unlike *I*_CaL_, which increases with depolarization up to 0 or +10 mV and reactivates regeneratively. Whether or not there was a contribution from *I*_Na/Ca_, it is clear that the electrical effect alone of widening the *I*_CaL_ window was sufficient, if not necessary, to generate EADs. These EADs were produced, in rabbit and human atrial myocytes, by symmetrical widening *I*_CaL_ window by simultaneously and equally shifting *V*_0.5_ of activation (negatively) and inactivation (positively). However, we found a strong EAD‐generating effect also resulted from shifting activation *V*_0.5_ alone (i.e. with asymmetrical‐widening). Because this effect was stronger than with shifting inactivation *V*_0.5_ alone, it suggests that the ability of a reactivating *I*_CaL_ to generate EADs is more dependent on a larger *I*_CaL_ arising from more negative potentials (in line with holding potential‐dependence of *I*_CaL_) (Li & Nattel, 1997) than on a relatively small *I*_CaL_‐increase permissible from shifting the inactivation curve towards values around the peak for *I*_CaL_ (0 or +10 mV).

Each of the responses to window widening, whether a single EAD or multiple EAD oscillations followed by normal repolarization, a single EAD or multiple EADs followed by repolarization failure, or indeed repolarization failure with no EAD but rather a non‐oscillating voltage signal (typically at *V*_m_ of the *I*_CaL_ window centre), can be considered in terms of a ‘Hopf bifurcation’ model of EADs (Qu *et al*. 2013). This model features analogous oscillations and ‘basin of attraction’ equilibrium states, including repolarization failure and ‘bi‐stable’ states of MDP‐alternans. The model emphasizes the major influence of *I*_CaL_ in non‐linear dynamical processes necessary for EADs. It also highlights, however, the necessity of highly complex interactions between numerous ion currents and their gating variables for EADs to occur. This re‐enforces the utility of dynamic clamping for studying EAD mechanisms because such complex interactions, and those not yet discovered, occur physiologically in the myocyte.

Cardiomyocytes require isolation for measuring ion currents or dynamic clamping APs, but this removes the syncytial current sink that occurs *in vivo*. This sink should be expected to overcome the repolarization failure observed to follow many of the cellular EADs here, and also perhaps to dampen the EADs themselves. However, the inward current producing these cellular events would still influence net source‐to‐sink ratio in the direction of APD‐increase and EAD‐generation. Whether that could contribute to arrhythmias in tissues is under debate. Weiss *et al*. (2010) propose that, although thousands of adjacent cells need synchronized EADs to produce a threshold propagating premature AP, this synchronization can occur naturally from the interaction between the divergent influence of chaos driving irregular EADs and the convergent influence of electrotonic coupling via gap junctions. Synchronization of chaotic EADs could also amplify APD‐dispersion, forming ‘EAD islands’ next to regions without EADs, creating unidirectional conduction block and reentry. This self‐organizing system could simultaneously create trigger and substrate for reentry even when the tissue is homogeneous, although they would develop more rapidly in heterogeneous tissue (e.g. fibrosed, aged atrium). Coupling between myocytes and myofibroblasts could also aid EAD production, demonstrated by dynamic clamping rabbit ventricular myocytes with an analogous virtual gap junction current (Nguyen *et al*. 2012).

To test whether *I*_CaL,D‐C_ window narrowing could suppress or prevent EADs, we induced EADs either by increasing the conductance of an injected *I*_CaL,D‐C_ having a normal window (as produced, for example, by adrenergic stimulation) (Workman, 2010) or, in myocytes then failing to generate EADs, adding the APD‐prolonging influence of K^+^ current‐block from 4‐AP (Workman *et al*. 2000). These interventions should promote EADs by increasing reactivating current amplitude and dwell‐time, respectively (January & Riddle, 1989; Fozzard, 1992; Devenyi *et al*. 2017). The *I*_CaL,D‐C_ window narrowing intervention then applied, comprising a simultaneous equal shift of *V*_0.5_ of *I*_CaL,D‐C_ activation (positively) and inactivation (negatively), favoured shifting activation over inactivation in the window region itself, resulting in a rather asymmetrical window narrowing. This abolished all EADs, whether single, multiple, or preceding repolarization failure, presumably by affecting the portion of the window region (the left ‐activation ‐limb) shown above to be most sensitive for EAD production; preventing *I*_CaL_ reactivation. Although window *I*_CaL_ characteristics are determined partly by parameters additional to *V*_0.5_ of activation and inactivation curves, previous studies (Madhvani *et al*. 2011; Madhvani *et al*. 2015) on EADs in rabbit ventricle support the view that it is the altering of these *V*_0.5_ values, rather than the slopes of these curves or the kinetics of current activation or decay, which suppress EADs. EAD suppression from dynamic clamp reduction of a non‐inactivating ‘pedestal’ *I*_CaL_ (increased by H_2_O_2_) was also shown (Madhvani *et al*. 2015), which is a component of *I*_CaL_ that we did not detect or model in atrial myocytes.

Pathological changes to window *I*_CaL_ have been reported, in the ventricle. In dogs, chronic atrioventricular block‐induced hypertrophy was associated with window *I*_CaL_‐widening and EADs under β‐adrenergic stimulation (Antoons *et al*. 2007). A genetic mutation in a patient with Timothy syndrome, a rare multigenetic disorder including QT‐prolongation, may also cause window *I*_CaL_‐widening, APD‐increase and EADs (Boczek *et al*. 2015). In atrium, although *I*_CaL_ is markedly altered in terms of peak current density (reduced) and open probability (increased) in human chronic AF (i.e. electrophysiological remodelling) (Klein *et al*. 2003; Workman *et al*. 2008; Heijman *et al*. 2014), there are no reports, to our knowledge, of pathological changes in window *I*_CaL_ voltage‐dependence. Enhancement of atrial window *I*_CaL_ (e.g. from a pathological widening of the window region and/or an increase in *I*_CaL_ preferentially in the window region) is unlikely to be a mechanism contributing to EAD production in clinical AF. However, it should be recognized that a therapeutically effective anti‐AF target does not require to be one that is pathologically altered, as evidenced by the two most effective anti‐AF drugs (amiodarone and flecainide), which are not considered to be effective by opposing the most prominent pathological ion current alterations (namely decreased *I*_CaL_ and *I*_TO_, and increased *I*_K1_) (Workman *et al*. 2011). Furthermore, a drug may be (and may be intended to be) more effective in un‐remodelled atrium (as investigated in the present study); for example, in the treatment of new‐onset or paroxysmal AF.

We propose that narrowing of a non‐enhanced (and non‐remodelled) *I*_CaL_ window may be considered as a potential therapeutic mechanism to inhibit atrial EADs caused by any primary mechanism, because of the likely prominent contribution ultimately (secondarily) of window *I*_CaL_ reactivation to such EADs. For example, *I*_CaL_ reactivation could occur with a sufficiently large increase in AP plateau duration, such as could result from adrenergic stimulation of *I*_CaL_ (Workman, 2010) (hence the present investigation of increasing *I*_CaL,D‐C_), K^+^ current reduction or inhibition (Workman *et al*. 2001) (hence our use of 4‐AP) or early (2–3 weeks) heart failure (Stambler *et al*. 2003; Yeh *et al*. 2008), all of which have potential relevance to AF. However, it must be recognized that dynamic clamping can assess only electrical (i.e. in the absence of CICR) effects of changing the current, and this is true for increasing *I*_CaL,D‐C_ conductance to provoke EADs, as well as for narrowing window *I*_CaL_ to prevent EADs. Alternatives to increasing *I*_CaL,D‐C_ could be considered in future studies, such as oxidative stress (Sato *et al*. 2009; Madhvani *et al*. 2011; Zhao *et al*. 2012; Madhvani *et al*. 2015) or hypokalaemia (Madhvani *et al*. 2011; Madhvani *et al*. 2015), as used previously in the ventricle.

The potential clinical therapeutic utility of window *I*_CaL_ narrowing is supported by recent reviews (Karagueuzian *et al*. 2017; Ortega *et al*. 2018) and based on the expectation (from modelling: Madhvani *et al*. 2011; Madhvani *et al*. 2015; Karagueuzian *et al*. 2017) that this intervention is unlikely to substantially affect [Ca^2+^]_i_ and thus excitation‐contraction coupling. By contrast, traditional CCBs (e.g. verapamil, dihydropyridines), although blocking window *I*_CaL_ to some extent, block peak and window *I*_CaL_ indiscriminately (i.e. with no preferential effect in the window voltage range). Inhibition of normally activating (not re‐activating) *I*_CaL_ (i.e. peaking at voltages largely positive to the window region), depresses excitation–contraction coupling, and thus limits therapeutic value, particularly in patients with compromised cardiac function. Future studies of any candidate window *I*_CaL_ narrowing drug(s) should assess their effects on all aspects of atrial excitation–contraction coupling, and using a variety of EAD‐promoting interventions. These could include, as studied in ventricle, [Ca^2+^]_i_‐overload from isoprenaline and/or BayK 8644 (January & Riddle, 1989; Zhao *et al*. 2012), as well as oxidative stress, hypokalaemia or heart failure.

The present dynamic clamp study of *I*_CaL_, the first to our knowledge in atrial cells, has identified a potentially anti‐arrhythmic (in isolated cells, at least) ion current (electrical component) mechanism of action. If new drugs could be developed (or existing ones identified or modified) that preferentially target the *I*_CaL_ window region, by shifting voltage‐dependent activation and/or inactivation curves with the result of narrowing the *I*_CaL_ window, without substantially affecting [Ca^2+^]_i_ and thus excitation–contraction coupling, and with minimal adverse effects, then these drugs could be much needed candidates for testing for anti‐AF action, *in vitro* and *in vivo*.

## Additional information

### Competing interests

The authors declare that they have no competing interests.

### Author contributions

All experiments were performed in the Sir James Black Laboratories, Institute of Cardiovascular & Medical Sciences, College of Medical, Veterinary & Life Sciences, University of Glasgow, Glasgow, UK. SK, PS, JD, MAC, RCM, GLS and AJW were responsible for the conception or design of the work. SK, PS, JD, MAC, RCM, GLS and AJW were responsible for the acquisition, analysis or interpretation of data. SK, PS, JD, MAC, RCM, GLS and AJW were responsible for drafting the work or revising it critically for important intellectual content. All authors approved the final version of the manuscript, agree to be accountable for all aspects of the work in ensuring that questions related to the accuracy or integrity of any part of the work are appropriately investigated and resolved, and all persons designated as authors qualify for authorship, and all those who qualify for authorship are listed.

### Funding

This work was supported by: British Heart Foundation Project Grant (PG/13/31/30156) (AJW, JD, GLS); Medical Research Council Strategic Skills Fellowship (MR/M014967/1) (MAC).
